# Promoting patient comprehension of relevant health information

**DOI:** 10.1186/s13584-018-0250-z

**Published:** 2018-09-18

**Authors:** Gary L. Kreps

**Affiliations:** Center for Health and Risk Communication, George Mason University, 4400 University Drive, MS 3D6, Fairfax, Virginia, 22030 USA

**Keywords:** Health communication, Health literacy, Patient comprehension, Teach back method

## Abstract

Patient understanding of health care recommendations provided by health care professionals is essential to enabling active and informed patient participation in care. Unfortunately, evidence suggests that patients often seriously misunderstand relevant health information provided to them, leading to errant patient decisions about their care. This commentary examines key communication factors that influence patient understanding and argues for a comprehensive approach to assessing and promoting patient comprehension.

## Commentary

Promoting patient comprehension of health care recommendations is essential to the effective delivery of health care. Yet, patients often misunderstand relevant health information provided to them by health care practitioners, leading to dangerous errors in following recommended care plans [1–3]. Promoting patient understanding of diagnostic and prognostic information is a complex task due to numerous potential barriers to achieving high levels of understanding, including limitations in both the levels of patient health literacy and in health care providers’ abilities to communicate complex health information clearly [4–6].

Health literacy is not just a trait that is rooted in patients’ levels of education, intelligence, and communication competence, but is also a state that is influenced by the physical, cognitive, and emotional situations individuals experience [4]. The stresses of confronting being ill can have a very negative influence on levels of health literacy, making it difficult for health care providers to explain complex health issues to patients. Moreover, the process of explaining complex health issues is challenging for many health care providers, who need to communicate with culturally, relationally, and situationally sensitive language and examples that patients can understand and actively seek feedback from patients to assess their levels of understanding [5, 6]. It is important for health researchers to carefully examine the processes of promoting patient comprehension of health information to increase understanding about the intricacies of consumer-provider health communication and to improve effective dissemination of relevant health information.

I commend Shiber, Zuker-Herman, Drescher, and Glezerman (2018) for embarking on their important research evaluating patient understanding of care plans communicated to patients while being seen in a hospital Emergency Department [7]. Emergency Department visits are especially challenging sites for effective consumer-provider communication due to the immediate time and attentional demands placed on health care providers who address health emergencies, as well as the fragile physical, mental, and emotional conditions of many patients receiving emergency care [5, 8]. Promoting understanding of care plans is an essential part of Emergency Department care that enables patients to make informed decisions about their care and to follow care plan recommendations.

While the Shiber, Zuker-Herman, Drescher, and Glezerman (2018) study is a good start for examining this important issue, it does not appear to go far enough to fully assess patients’ levels of comprehension of their care plans [7]. The researchers asked patients how well they understood the care plans described to them, without checking the accuracy of patients’ comprehension of the health information provided. It is common for patients to report understanding relevant health information provided during health care visits, while actually being very poorly informed about their diagnoses, prognoses, and care plans [3, 5, 6]. Patients may think that they understand the health information provided to them, when not really fully comprehending relevant information. Patients are also likely to claim falsely to understand health care recommendations as an attempt to maintain face, appear to be competent, and to show that they are in-charge of their care [5, 6]. Patients may be embarrassed to report that they are confused and do not understand relevant health information. This can lead both health care providers and researchers to over-estimate levels of patient understanding of the health information provided to them.

National studies about medication adherence and international studies of health information dissemination have found that patients are often terribly misinformed about their health conditions, despite often believing that they are well informed [3, 9–12]. These studies not only asked patients how much health information about their conditions that they understood, but also objectively tested the accuracy of their understanding about health conditions and treatment plans. Even when patients reported understanding their health conditions and treatment plans, these studies found that many of these patients were badly misinformed, did not report accurate understanding of their diagnoses, did not understand their treatment plans, and due to these information deficiencies did not faithfully follow their health care plan recommendations, often undermining the quality of their health care. For example, one national study in the US found that more than 50% of patients with serious chronic diseases were not taking the prescribed medications, often because they misunderstood information about their diagnoses, treatment plans, and the importance of their taking their medications [3]. Failure for many of these chronic disease patients to take their prescribed medications could be life-threatening.

Future research should expand upon the Shiber, Zuker-Herman, Drescher, and Glezerman study by implementing additional measures to fully assess patient understanding of their treatment plans [7]. I suggest employing objective tests of levels of patient understanding by asking patients to fully explain their diagnoses and treatment plans to the best of their abilities. Researchers can then compare these respondent explanations to the patients’ actual diagnoses and treatment plans. This is a research application of the teach-back communication method, where providers ask their patients to explain the health information provided to them; research about the use of the teach back method found that this communication strategy can improve patient comprehension of health information provided to them in emergency care [13]. Researchers can also utilize other methods to assess understanding, including employing self-report measures, observational research strategies, and archival analysis of health records to increase confidence in research findings about patient comprehension [14, 15].

## Conclusions

It is important to recognize the complexities of effective communication between health care providers and their patients, and to make certain that studies of patient comprehension of relevant health information are valid. The Shiber, Zuker-Herman, Drescher, and Glezerman study provides a good starting point for programmatic research about patient comprehension of relevant health information by examining the levels of patient confidence in understanding their care plans [7]. The next step in this program of research is to examine patients’ actual levels of understanding. It will be interesting to compare patient estimates of comprehension about their care plans with their actual understanding about these care plans. This program of expanded patient comprehension research has great potential to guide development of important health communication programs, policies, and tools that can enhance quality of care and improve health outcomes.
